# Poor association of allergen‐specific antibody, T‐ and B‐cell responses revealed with recombinant allergens and a CFSE dilution‐based assay

**DOI:** 10.1111/all.12661

**Published:** 2015-07-28

**Authors:** J. Eckl‐Dorna, R. Campana, R. Valenta, V. Niederberger

**Affiliations:** ^1^ Department of OtorhinolaryngologyMedical University of ViennaViennaAustria; ^2^ Division of ImmunopathologyDepartment of Pathophysiology and Allergy ResearchCenter for Pathophysiology, Infectiology and ImmunologyMedical University of ViennaViennaAustria

**Keywords:** allergy, B cell, carboxyfluorescein‐diacetate‐succinimidylester, proliferation, T cell

## Abstract

**Background:**

The adaptive immunity underlying allergy comprises two components, the allergen‐specific antibody (i.e. IgE, IgG) and the T‐cell response. These two components are responsible for different disease manifestations and can be targeted by different therapeutic approaches. Here, we investigated the association of allergen‐specific antibody and T‐ as well as B‐cell responses in pollen‐allergic patients using recombinant (r) major birch pollen allergen rBet v 1 and major timothy grass pollen allergen rPhl p 5 as defined antigens.

**Methods:**

Allergen‐specific IgE and IgG antibody responses were determined by ELISA, and allergen‐specific T‐ and B‐cell responses were measured in peripheral blood mononuclear cells using a carboxyfluorescein‐diacetate‐succinimidylester (CFSE) dilution assay.

**Results:**

CFSE staining in combination with T‐cell‐ and B‐cell‐specific gating allowed discriminating between allergen‐specific T‐cell and B‐cell responses. Interestingly, we identified patients where mainly T cells and others where mainly B cells proliferated in response to allergen stimulation. No association between the level of allergen‐specific Ig responses and B‐ or T‐cell proliferation was observed.

**Conclusion:**

Purified recombinant allergens in conjunction with CFSE staining allow the dissection of allergen‐specific B‐ and T‐cell responses. The dissociation of allergen‐specific antibody, and B‐ and T‐cell responses may explain the occurrence of selective IgE‐ and T‐cell‐mediated manifestations of allergic inflammation and may be important for the development of diagnostic and therapeutic strategies selectively targeting B cells and T cells.

**Abbreviations**
^3^H: tritiated
7‐AAD: 7‐amino‐actinomycin‐D
ABTS: 2,2′‐azino‐bis(3‐ethylbenzothialzoline‐6‐sulphonic acid) diammonium salt
APT: atopy patch testing
BSA: bovine serum albumin
CFSE: carboxyfluorescein‐diacetate‐succinimidylester
Cpm: counts per minute
FCS: foetal calf serum
H_2_O_2_: hydrogen peroxide
Ig: Immunoglobulin
PBMCs: peripheral blood mononuclear cells
PC: phycoerythrin cyanine
R: recombinant
SIT: allergen‐specific immunotherapy

Allergy is an important health problem affecting more than 20% of the population in industrialized countries 1, 2. The adaptive allergen‐specific immune response comprises the allergen‐specific antibody (i.e. IgE, IgG) and the allergen‐specific T‐cell response 3. Immediate‐type responses usually occur within the first 30 min after allergen contact and are mediated by the cross‐linking of IgE bound to the high‐affinity receptor for IgE (FcεRI) on the surface of basophils and mast cells. The release of inflammatory mediators, proinflammatory cytokines and proteases causes the typical symptoms of immediate‐type reactions 4.

By contrast, allergen‐specific T cells play an important role in the regulation of the allergic immune response and in chronic allergic inflammation 5. Clinical studies strongly suggest that T‐cell‐mediated responses can occur independently of IgE‐mediated responses 6, 7, 8, 9. For example, upon intradermal administration of short overlapping peptides derived from the major cat allergen Fel d 1, late asthmatic reactions in the absence of early symptoms occurred 6. These peptides were too short to bind IgE but fully retained their T‐cell epitopes. Similarly, recombinant hypo‐allergens without IgE reactivity but intact T‐cell epitopes have been observed to cause T‐cell‐mediated side‐effects in the course of allergen‐specific immunotherapy (SIT) and by atopy patch testing (APT) 9, 10. Ideally, newly developed allergy vaccines should avoid both B‐ and T‐cell‐mediated side‐effects. Therefore, a thorough analysis and understanding of allergen‐specific B‐ and T‐cell responses is of great importance for the design of new therapeutic approaches and for the development of biomarkers to monitor SIT 11.

During the last years, MHC class II peptide tetramers have been successfully used to assess allergen‐specific T‐cell responses in allergic and nonallergic individuals during and outside pollen seasons 12, in allergic patients suffering from seasonal *vs* perennial allergies 13 and in the course of SIT 14. MHC class II peptide tetramers were found to be valuable tools to study qualitatively and quantitatively allergen‐specific T‐cell responses. However, this approach has also some important limitations, amongst them that only certain high‐affinity T‐cell epitopes can be studied and that the approach is limited to subjects with certain MHC background 15.

Here, we demonstrate that the combined use of highly purified recombinant allergens with a carboxyfluorescein‐diacetate‐succinimidylester (CFSE) dilution assay 16 using selective T‐cell and B‐cell staining allows to discriminate allergen‐specific T‐cell from B‐cell responses directly in cultured peripheral blood mononuclear cells (PBMCs) from allergic patients. The approach did not require a preselection of patients or the use of selected allergen‐specific T‐cell epitopes. Interestingly, we found that in some patients, B cells are more prone to respond to allergen stimulation, whereas in others T cells proliferated upon allergen stimulation *in vitro*. In addition, we found that there was a dissociation of allergen‐specific T‐cell and antibody responses in allergic patients which may explain the occurrence of isolated IgE‐ and T‐cell‐mediated symptoms in allergic patients and which should be important for the development of selective immunotherapy strategies.

## Methods

### Reagents

PBMCs were cultured in Ultra Culture Medium (Lonza Group LTD, Basel, Switzerland) supplemented with 200 μM glutamine, 50 μM β‐Mercaptoethanol and 50 μM gentamicin (all Invitrogen Inc., Carlsbad, CA, USA). Ficoll and ^3^H‐thymidine were purchased from GE Healthcare (Buckinghamshire, UK). Recombinant allergens Bet v 1 (Endotoxin content: 0.072 EU/μg) and Phl p 5 (Endotoxin content: 0.003 EU/μg) were obtained from Biomay AG (Vienna, Austria) and dynabeads human T‐activator CD3/28 from Invitrogen Inc. For flow cytometry, the following reagents were used: anti‐CD3 PC7 (clone UCHT1), anti‐CD20 PC5 (clone HRC20), mouse IgG_1_ PC7, mouse IgG_2a_ PC5 and 7‐amino‐actinomycin‐D (7‐AAD) were purchased from Beckmann Coulter Inc. (Fullerton, CA, USA); fixable viability dye eFluor^®^ 780 and Mouse IgG_2a_ PC7 from eBioscience, Inc. (San Diego, CA, USA.); anti‐CD14 PC7 (clone M5E2) from BD Biosciences (San Jose, CA, USA); and CFSE from Invitrogen Inc. Anti‐human IgG as well as anti‐human IgE‐HRP were bought from BD Biosciences, ELISA plates from Nunc Maxisorp (Roskilde, Denmark) and bovine serum albumin (BSA) from PAA (Pasching, Austria). HRP‐labelled anti‐mouse IgG antibody was purchased from GE Healthcare and 2,2′‐azino‐bis (3‐ethylbenzothialzoline‐6‐sulphonic acid) diammonium salt (ABTS) and hydrogen peroxide (H_2_O_2_) from Sigma Aldrich (St. Louis, MO, USA).

### Patients, cell isolation and culture

Birch‐ and grass‐pollen‐allergic patients (*n* = 14) were included in this study after written informed consent was obtained from all patients before blood taking. This study was approved by the Ethical Committee of the Medical University of Vienna. Eleven patients were sensitized to both birch and grass pollen, two were allergic only to birch pollen (patient #8 and #9) and in one patient, it was not known whether he suffered from symptoms of grass‐pollen allergy in addition to birch pollen allergy (patient #14) (Table S1).

PBMCs were isolated from heparinized blood samples by Ficoll density gradient centrifugation. PBMCs – either unlabelled or labelled with CFSE (see below) – were cultured at a concentration of 2 × 10^5^ cells per well in 96‐well plates. Cells were either left unstimulated (medium control) or stimulated with human T‐cell activator (i.e. dynabeads containing anti‐CD3 and anti‐CD28, 15 μl of dynabeads/ml medium) or Bet v 1 or Phl p 5 at different concentrations (25 μg/ml and 5 μg/ml, and in some experiments also 0.5 μg/ml as indicated in the figures). Cells were analysed on day 7 if not otherwise indicated.

### ^3^H thymidine incorporation

Cultures were pulsed with ^3^H‐thymidine at 0.5 μCi/well for 16 h before harvest, and ^3^H‐thymidine incorporation was measured using a beta counter (MicroBeta TriLux; PerkinElmer, Waltham, MA, USA). Stimulation index (SI) was calculated as following: SI = counts per minute (cpm) of stimulated cells/cpm of unstimulated cells. A cut‐off for proliferation was set at a SI > 1.

### CFSE labelling and flow cytometry

CFSE labelling was performed as previously described 17. Briefly, PBMCs isolated from blood by Ficoll gradient were washed three times in PBS and incubated with CFSE (Invitrogen Inc.) in PBS (labelling concentration of 5 μM) for 10 min at 37°C. The labelling reaction was stopped by adding pure foetal calf serum (FCS) (PAA) for 5 min. Cells were then washed with medium and cultured as described above. Upon harvest, cells were washed, resuspended in PBS 1% BSA and stained with the respective antibodies, isotype controls and the viability dye on ice for 20 min. Flow cytometry was performed using a FC500 (Beckmann Coulter Inc., Fullerton, CA, USA). Thirty thousand events were acquired per sample and analysed with Flowjo Software (Treestar Inc., Ashland, OR, USA). If 30 000 events could not be recorded, absolute cell numbers were extrapolated to allow for the comparison of cell numbers in Tables S3 and S4.

A consecutive gating strategy – exemplified for T and B cells in Fig. S1 – was employed to identify proliferating T or B cells. First, lymphocytes were gated according to morphological criteria on a forward scatter/sideward scatter dot plot to exclude cell debris (Fig. S1A,E). Next, alive cells were selected by negative staining for the viability dye 7‐AAD (Fig. S1B,F), followed by gating on CD3‐positive cells to focus on T cells (Fig. S1C) and, in a separate experiment, by gating on CD20‐positive cells to focus on B cells (Fig. S1G). Proliferated T cells and B cells were identified by their low staining for CFSE (CFSE^low^ T cells, CFSE^low^ B cells), respectively (Fig. S1D,H). The percentages of proliferated T cells of total T cells and of proliferated B cells of total B cells were calculated as follows 18: percentage of proliferated T or B cells of total T or B cells = percentage of allergen‐stimulated proliferated T or B cells (CFSE^low^ stim CD3 or CD20‐positive cells/total stim CD3 or CD20‐positive cells) – percentage of unstimulated (i.e. medium alone) proliferated T or B cells (CFSE^low^ unstim CD3 or CD20‐positive cells/total unstim CD3 or CD20‐positive cells).

### ELISA

ELISA was performed as previously described 19. Briefly, ELISA plates were coated overnight at 4°C with either Bet v 1 or Phl p 5 (5 μg/ml) diluted in coating buffer (100 mM sodium carbonate‐bicarbonate). Then, plates were washed (PBS, 0.02% v/v Tween 20) and blocked with blocking buffer (PBS, 0.02% v/v Tween 20, 1% w/v BSA) for 2 h at room temperature. Plates were incubated overnight at 4°C with patient's sera diluted 1 : 5 (for IgE measurement) or 1 : 20 (for IgG measurement) in blocking buffer. For IgG measurement, anti‐human IgG antibody was applied at a dilution of 1 : 1000 in blocking buffer and incubated overnight at 4°C followed by a 2‐h incubation with HRP‐labelled anti‐mouse IgG antibody at a dilution of 1 : 2500 in blocking buffer. For IgE measurement, anti‐human IgE‐HRP was applied at a dilution of 1 : 1000 in blocking buffer for 2 h at room temperature. For detection, ABTS was used at a concentration of 1 mg/ml in phosphate‐buffered citrate (70 mM) and 0.1 μl/ml H_2_O_2_ (30%) was added. Plates were read at 405 nm (Victor3; PerkinElmer). Antibody levels correspond to OD values, which represent means of triplicate determinations ± SD.

### Statistical analysis

Correlation between different data sets was calculated using Spearman's ρ coefficient. Analyses were performed using SPSS software (version 20.0; IBM, New York, NY, USA).

## Results

### In allergic patients, B and T cells respond to a different extent to allergen stimulation as measured with a CFSE dilution‐based assay

As exemplified in Fig. S2A, T cells from birch‐ and grass‐pollen‐allergic patients (#7, Table S1) proliferated in response to Bet v 1 and Phl p 5 after 7 days and were identified by positive staining for anti‐CD3 and low CFSE staining. Commonly used protocols for ^3^H‐thymidine incorporation measure proliferation in PBMC cultures on days 6–7 after stimulation 20, 21, 22. To study whether this would be also a suitable time point for measurement of proliferation by CFSE, we stimulated PBMCs for different periods (3, 5 and 7 days) and assessed proliferation by CFSE staining and ^3^H‐thymidine incorporation (Fig. S2B). This experiment yielded comparable results when performed in two patients [#3 (Fig. S2B) and #7 (data not shown)]. Best proliferation with the CFSE dilution assay was observed on day 7 but not on day 3 and on day 5 with both allergens at each of the tested concentrations. Therefore, day 7 was defined as the optimal time point for the measurement of proliferation in PBMCs upon allergen challenge by CFSE.

**Figure 1:**
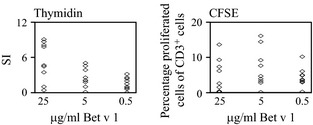
Proliferation of T cells in response to Bet v 1. PBMCs from nine allergic patients (#1–9) were stimulated with Bet v 1 at 25, 5 or 0.5 μg/ml (*x*‐axes). Proliferation of total PBMCs was assessed by ^3^H‐thymidine incorporation (left; *y*‐axes: stimulation indices SI), and proliferation of T cells was assessed by CFSE dilution (right; *y*‐axes: percentage proliferated cells of CD3^+^ cells).

We measured T‐cell proliferation by CFSE dilution assay in nine allergic patients to confirm the reliability of the test for the measurement of T‐cell proliferation in response to allergens. In these patients, proliferation was also performed using ^3^H‐thymidine incorporation as a standard readout of proliferation. CFSE‐labelled PBMCs were stimulated with Bet v 1 and Phl p 5 at concentrations of 0.5, 5 or 25 μg/ml. We observed proliferation with the CFSE dilution assay with all three concentrations; however, the highest percentage of proliferation was observed using 5 μg/ml of the respective allergens (Bet v 1 Fig. 1 and Phl p 5 Fig. S3A). When proliferation was measured by ^3^H‐thymidine incorporation, highest stimulation indices were observed with the highest concentration of allergen (i.e. 25 μg/ml).

**Figure 2:**
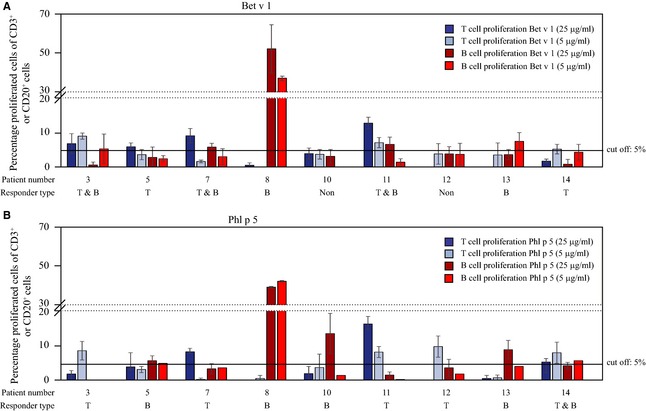
Proliferation of B and T cells in response to Bet v 1 and Phl p 5. Proliferation of T cells (blue) and B cells (red) (*x*‐axes) in response to 25 (dark colour) or 5 μg/ml (bright colour) of Bet v 1 (A: upper panel) or Phl p 5 (B: lower panel) was assessed in nine allergic patients (#3, 5, 7, 8, 10–14) by CFSE dilution experiments. Results are shown as percentage of proliferated cells of CD3^+^ or CD20^+^ cells respectively. To determine the responder type, a cut‐off of 5% was determined: For a ‘responder’, proliferation had to be above 5% in at least one of the two concentrations tested in the respective population (CD3^+^ or CD20^+^ cells). Results are displayed as mean values of triplicate measurements.

In the next step, we were interested to determine whether allergen stimulation can induce proliferation also in immune cells other than T cells present in PBMC cultures of allergic patients. For this purpose, we stimulated CFSE‐labelled PBMC cultures of nine allergic patients with two different concentrations of Bet v 1 or Phl p 5 (25 or 5 μg/ml) for 1 week and then stained them with the pan B‐cell marker anti‐CD20 or with anti‐CD14 for identification of monocytes. Most monocytes were dead after 1 week and were identified by positive staining for 7‐AAD. Thus, we could not measure proliferation in this subset due to the small number of CD14‐positive cells in the alive cell gate (data not shown). However, when we gated on CD20‐positive B cells, we observed proliferation in response to both Bet v 1 (Figs 2A and S4) and Phl p 5 (Figs 2B and S4). We also determined T‐cell proliferation in those patients (Fig. 2A,B) and identified three different responder types according to the preponderance of B‐ or T‐cell proliferation in response to allergen stimulation (Fig. 2, Tables S2–S4): B‐cell responder (Bet v 1: 2 patients, Phl p 5: 4 patients), T‐cell responder (Bet v 1: 2 patients, Phl p 5: 4 patients) and B‐ and T‐cell responder (Bet v 1: 3 patients, Phl p 5: 1 patient). In Bet v 1 stimulated cultures, 2 patients were classified as nonresponders.

**Figure 3:**
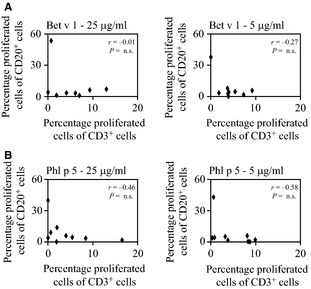
Correlation of allergen‐specific T‐ and B‐cell proliferation. Scatter plots of T‐ (*x*‐axes) and B (*y*‐axes)‐cell proliferations as measured by CFSE dilution in response to stimulation with 25 (left) or 5 (right) μg/ml of (A) Bet v 1 (B) Phl p 5. Results are displayed as percentage of proliferated cells of CD3^+^ or CD20^+^ cells, respectively. Experiments were performed in triplicates in nine allergic patients (#3, 5, 7, 8, 10–14), and the mean values are displayed.

We next assessed whether there was any association between the extent of B‐ and T‐cell proliferation in allergic patients upon allergen stimulation. As shown in Fig. 3, no relevant association was observed between B‐ and T‐cell proliferation regardless of the allergen or concentration used (Spearman's ρ correlation coefficient between −0.58 and −0.01, *P *= not significant).

These results demonstrate that both T and B cells of allergic patients proliferated to a different extent in response to allergen stimulation which can be discriminated by CFSE dilution assay but not by ^3^H‐thymidine incorporation.

### Poor association between allergen‐specific antibody levels and T‐ or B‐cell proliferation

**Figure 4:**
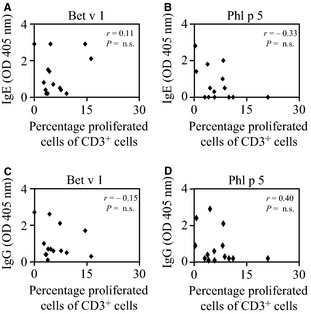
Correlation of allergen‐specific IgE and IgG levels with T‐cell proliferation. (A–D) Scatter plots of T‐cell proliferations (*x*‐axes) as measured by CFSE dilution in response to stimulation with 5 μg/ml (A and C) Bet v 1 or (B and D) Phl p 5 and allergen‐specific (A and B, *y*‐axes) IgE or (C and D, *y*‐axes) IgG. Experiments were performed in triplicates in 14 patients (#1–14), and the mean values are displayed.

Next, we aimed to assess whether the extent of T‐ or B‐cell proliferation as measured by CFSE dilution is associated with the levels of allergen‐specific antibodies (i.e. IgE, IgG) in allergic patients. Bet v 1‐ and Phl p 5‐specific IgE was determined by ELISA (19). First, we determined whether there was an association between the extent of allergen‐specific T‐cell proliferation and serum IgE levels. As shown in Fig. 4A, poor association was observed between allergen‐specific IgE levels and allergen‐specific T‐cell proliferation regardless of the allergen used (Spearman's ρ correlation coefficient between −0.33 and 0.11, *P* = not significant). Also allergen‐specific T‐cell proliferation upon stimulation with lower or higher concentrations of allergen and antibody levels were not related (Spearman's ρ correlation coefficient between −0.38 and 0.51, data not shown). There was also no correlation observed between allergen‐specific IgG levels and allergen‐specific T‐cell proliferation (Fig. 4B, Spearman's ρ correlation coefficient between −0.15 and 0.40, *P* = not significant). Interestingly, patients were identified with high allergen‐specific T‐cell responses with relatively low antibody responses (e.g. patient 3 and 5 for Bet v 1 and patient 3 and 12 for Phl p 5) (Table S2) and others with high allergen‐specific antibody responses but little specific T‐cell responses (e.g. patient 8 for Bet v 1; patient 13 for Phl p 5) demonstrating the dissociation of allergen‐specific antibody and T‐cell responses.

**Figure 5:**
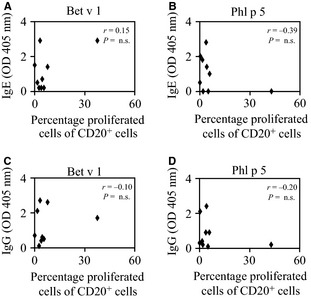
Correlation of allergen‐specific IgE and IgG levels with B‐cell proliferation. (A–D) Scatter plots of B‐cell proliferations (*x*‐axes) as measured by CFSE in response to stimulation with 5 μg/ml (A and C) Bet v 1 or (B and D) Phl p 5 and allergen‐specific (A and B, *y*‐axes) IgE or (C and D, *y*‐axes) IgG. Experiments were performed in triplicates in nine allergic patients (#3, 5, 7, 8, 10–14), and the mean values are displayed.

Next, we determined the association of allergen‐specific B‐cell proliferation and IgE and IgG levels. As shown in Fig. 5, no correlation could be observed between allergen‐specific B‐cell proliferation and allergen‐specific IgE (Spearman's ρ correlation coefficient between −0.39 and 0.15) or IgG levels (Spearman's ρ correlation coefficient between −0.20 and −0.10). Also here, we identified patients with high allergen‐specific B‐cell responses with relatively low antibody responses (e.g. patient 3 for Bet v 1 and patient 8 for Phl p 5) and others with high allergen‐specific antibody responses but little specific B‐cell responses (e.g. patient 10 for Bet v 1 and patient 7 and 11 for Phl p 5). Thus, no association was observed between allergen‐specific B‐ or T‐cell proliferation *in vitro* and serum IgE or IgG levels.

## Discussion

In the present study, we used highly purified recombinant pollen allergens to dissect allergen‐specific T‐cell, B‐cell and antibody responses in allergic patients. Allergen‐specific T‐cell‐ and B‐cell‐proliferative responses were studied with a CFSE dilution assay gating on T cells or B cells, respectively. We found that PBMCs from allergic patients contained not only T cells which proliferated in response to allergen exposure but also B cells. This finding suggests that the CFSE dilution assay when combined with appropriate gating strategies has an important advantage compared with conventional PBMC proliferation assays based on ^3^H‐thymidine incorporation assays which cannot discriminate T‐cell from B‐cell proliferation in PBMC cultures. Interestingly, patients were identified with high allergen‐specific antibody responses without detectable T‐cell responses and others with very low allergen‐specific antibody responses but specific T‐cell responses indicating a dissociation of allergen‐specific antibody and T‐cell responses. This observation was also true when allergen‐specific IgE and IgG levels were correlated with allergen‐specific T‐cell responses in each of the tested patients. Furthermore, no association between allergen‐specific B‐cell proliferation and allergen‐specific serum Ig levels was observed.

In the present study, we observed T‐cell proliferation using the CFSE dilution assay on day 7, whereas proliferation of cells employing ^3^H‐thymidine incorporation was already observed on day 5 of culture. This discrepancy may be explained by the different parameters measured by the two assays: ^3^H‐thymidine assay measures DNA replication, which precedes actual cell division as measured by the CFSE assay. This may explain for the different results observed on days 5 and 7 for the CFSE and ^3^H‐thymidine assay.

Previous studies comparing ^3^H‐thymidine incorporation in PBMC cultures stimulated with allergen extract or natural allergen preparations with specific IgE antibody levels provided controversial results. While certain studies suggested that there is no correlation between allergen‐specific IgE and T‐cell responses 23, 24, 25, others reported a good correlation between specific IgE levels and T‐cell proliferation in allergic individuals 26. It is quite possible that the discrepant findings in these earlier studies are due to several important confounding factors. First of all, allergen extracts contain a number of different allergens as well as a high number of undefined nonallergenic proteins. It is therefore impossible to discriminate between allergen‐specific T‐cell responses and T‐cell responses specific for nonallergenic components. Second, it has been shown that allergen extracts contain potent immunomodulatory factors 27 which may strongly influence lymphocyte proliferation results. Third, natural allergen preparations are known to contain various allergen isoforms with different IgE reactivity and T‐cell‐stimulatory capacities 20. A further technical limitation of the previous studies was that they used ^3^H‐thymidine incorporation in PBMC cultures as readout for T‐cell proliferation 23, 25, 26. However, as shown here and as previously observed in autoimmune cells 29 and in PBMCs from grass–pollen‐allergic donors 29, both B and T cells may respond to stimulation with proliferation and thus thymidine incorporation does not reflect exclusively T‐cell responses. Finally, it must be borne in mind that not all of the allergen‐specific T cells are directly involved in the induction of IgE responses. One must therefore also take other antibody isotypes into consideration when comparing allergen‐specific T‐cell and antibody responses. As allergic patients besides producing allergen‐specific IgE also mount allergen‐specific IgG but little or no allergen‐specific IgA or IgM responses 30, 31, we have included also specific IgG but found no correlation with T‐cell responses.

The dissociation of allergen‐specific antibody and T‐cell responses observed by us may be important because it explains the occurrence of selective IgE‐ and T‐cell‐mediated manifestations of allergic inflammation in patients upon allergen exposure. Our findings also would fit to data obtained in murine models of allergy and from HIV‐infected allergic patients suffering from AIDS showing that the secondary allergen‐specific IgE response does not require T‐cell help 32, 33.

Furthermore, we observed poor association of allergen‐specific serum Ig titres with allergen‐specific B‐cell proliferation. It has previously been shown that the blood contains IgE‐producing cells 34, which have been identified as plasma cells 35. However, blood‐derived plasma cells accounted only for a small percentage of IgE found in the circulation and it is therefore assumed that IgE is either produced locally in target organs of allergy such as the nasal mucosa 36 and the respiratory tract 37 or in the bone marrow 38. This may explain for the observed lack of correlation between allergen‐specific serum IgE titres and allergen‐specific blood‐derived B‐cell proliferation.

Nowadays, multicolour flow cytometry using several lasers and multiple channels has become widely available and opens the way towards assessing various parameters in addition to proliferation. In this respect, CFSE can be used in combination with MHCII tetramer staining to identify allergen‐specific clones of interest amongst the proliferating population of T cells 15. It is, however, equally possible to use allergen‐derived peptides in combination with the CFSE dilution assays to study not only allergen but also epitope‐specific T‐cell responses in patients, for example to identify frequently recognized T‐cell epitopes to be used in T‐cell tolerance induction approaches. Advantages of the CFSE dilution assay are that there is no limitation from the MHC background of the patients or a necessity to identify high‐affinity T‐cell epitopes as in MHCII tetramer approaches. However, when using fluorescent probes for labelling of cells it needs to be borne in mind that they may also influence various cell functions due to interaction of the dye with different cellular components. As such, CFSE has been reported to have a negative effect on lymphocyte function, viability and proliferation if used at high concentrations 39, 40. Thus, to avoid adverse effects, CFSE should be used at the lowest possible concentration.

Moreover, CFSE dilution assays might be applied for immune monitoring of the success of immunotherapy. Finally, it may provide a useful tool for future functional studies of cellular responses to allergen to facilitate the development of new immunotherapy strategies, which selectively target T‐cell and B‐cell responses in allergy.

## Funding

Supported by grants F4613 and F4605 from the Austrian Science Fund (FWF).

## Conflict of interest

Rudolf Valenta has received research grants from Biomay AG, Vienna, Austria, Thermofisher, Uppsala, Sweden and Fresenius Medical Care, Bad Homburg, Germany and serves as consultant for these companies.

## Supporting information


**Figure S1.** Gating strategy.

**Figure S2.** Kinetics of T‐cell proliferation as measured by CFSE dilution.

**Figure S3.** Proliferation of T cells in response to Phl p 5.

**Figure S4.** B‐cell proliferation as measured by CFSE dilution.

**Table S1.** Demographic and clinical characterization of pollen‐allergic patients.

**Table S2.** Percentages of proliferated cells of B and T cells in PBMCs in response to allergens Bet v 1 and Phl p 5 and allergen‐specific IgE and IgG levels.

**Table S3.** Comparison of total proliferated cells, T cells and proliferated T cells in response to allergens Bet v 1 and Phl p 5.

**Table S4.** Comparison of total proliferated cells, B cells and proliferated B cells in response to allergens Bet v 1 and Phl p 5.
